# Detecting missing teeth on PMCT using statistical shape modeling

**DOI:** 10.1007/s12024-023-00590-w

**Published:** 2023-03-09

**Authors:** Dana Rahbani, Barbara Fliss, Lars Christian Ebert, Monika Bjelopavlovic

**Affiliations:** 1 Graphics and Vision Research Group (GraVis), University of Basel, Basel, Switzerland; 2 Institute of Forensic Medicine, University Hospital of Mainz, Mainz, Germany; 3 3D Center Zurich, Zurich Institute of Forensic Medicine, University of Zurich, Zurich, Switzerland; 4 Department of Prosthodontics and Materials Science, University Medical Center Mainz, Augustusplatz 2, 55131 Mainz, Germany

**Keywords:** Statistical shape model, Identification, Mandible, Teeth

## Abstract

The identification of teeth in 3D medical images can be a first step for victim identification from scant remains, for comparison of ante- and postmortem images or for other forensic investigations. We evaluate the performance of a tooth detection approach on mandibles with missing parts or pathologies based on statistical shape models. The proposed approach relies on a shape model that has been built from the full lower jaw, including the mandible and teeth. The model is fitted to the target, resulting in a reconstruction, in addition to a label map that indicates the presence or absence of teeth. We evaluate the accuracy of the proposed solution on a dataset consisting of 76 target mandibles, all extracted from CT images and exhibiting various cases of missing teeth or other cases, such as roots, implants, first dentition, and gap closure. We show an accuracy of approximately 90% on the front teeth (including incisors and canines in our study) that decreases for the molars due to high false-positive rates at the wisdom teeth level. Despite the drop in performance, the proposed approach can be used to obtain an estimate of the tooth count without wisdom teeth, tooth identification, reconstruction of the existing teeth to automate measurements taken as part of routine forensic procedures, or prediction of the missing teeth shape. In comparison to other approaches, our solution relies solely on shape information. This means it can be applied to cases obtained from either medical images or 3D scans because it does not depend on the imaging modality intensities. Another novelty is that the proposed solution avoids heuristics for the separation of teeth or for fitting individual tooth models. The solution is therefore not target-specific and can be directly applied to detect missing parts in other target organs using a shape model of the new target.

## Introduction

The identification of decomposed or mutilated bodies is one of the main tasks performed by forensic professionals all over the world on behalf of legal authorities. In situations that deal with a large number of fatalities, such as natural disasters (e.g., Tsunami in South Asia 2004 and flood catastrophe in Western Germany 2021), terrorist attacks, or traffic accidents, rapid and secure techniques are needed to identify the deceased.

As dentition is unique in every individual, it is a secure method of identification and one of the primary three identifiers according to INTERPOL standards. Furthermore, human teeth are the hardest substances in the body, which makes them likely to survive in cases of decomposition or accidents [1, 2]. As shown during the Tsunami in 2004, forensic odontology was a major component of identifying the corpses [3, 4] by comparing antemortem and postmortem data (presence of dental fillings, endodontic treatments, crowns or bridges, the presence of malocclusions or dental fractures, and radiographs). This identification process was mostly accomplished by comparing dental records or radiographs with the actual dentition of the deceased and/or postmortem radiographs, which as decomposition continued was not always a straightforward task to do. In the worst case, the jaw must be dissected [3]. These methods are very time-consuming and can be an inhibiting factor when dealing with mass fatalities.

Postmortem computed tomography (PMCT) is well established as a routine diagnostic tool in a number of forensic medicine institutes worldwide. It was first applied to a disaster victim identification (DVI) scenario on a regular basis during bushfires in Australia in 2009 [5, 6]. The incorporation of PMCT scans is recommended as a useful tool in DVI missions [7].

Dental identification using postmortem computed tomography is established in everyday casework, and its potential for use in DVI scenarios is obvious. Studies have shown that the accuracy of dental postmortem CT can be compared to those of postmortem radiographs [8–10]. However, it must be pointed out that the main limitation of using PMCT for dental identification is the appearance of streak artifacts due to metallic dental work, which can be reduced using an extended CT scale.

To adapt and improve this method of dental identification on PMCT, particularly to accelerate the process in case of fatalities with a high number of victims, we follow the approach of introducing machine learning to forensics, as automation is the key to reducing errors and increasing the speed of the identification. The aim of this study is to automate the process of identifying and labeling missing teeth in the mandible by using statistical shape modeling (SSM).

An approach, followed by Miki et al. [11], uses deep convolutional neural networks on cone beam CT data, which is not as commonly used and available in forensic institutes as normal multislice CT. An accuracy of over 80% was achieved for correctly identifying the following teeth: central incisor; lateral incisor; canine; first and second premolar; and first, second, and third molar. A different approach was followed by Momeni and Aghaeizadeh [12] and Hosntalab et al. [13], who used wavelet descriptors for automated teeth recognition and for detecting missing teeth [12, 13]. They reached an accuracy of 95% in teeth recognition in the maxilla and the mandible. statistical shape modeling was previously used by Duy et al. [14] to differentiate between existing or missing teeth of the maxilla [14, 15]. A maxilla SSM was first fitted to the target, after which the positions of planes that separate the maxilla into tooth regions were predicted. The intensity histograms between the planes were then used to determine whether a tooth was missing.

Contrary to the abovementioned studies, we use an SSM of both the mandible and teeth and hypothesize that we can label the presence or absence of individual teeth based on shape reconstruction. This is achieved by solving two problems simultaneously: the robust reconstruction problem, which aims to reconstruct the full mandible with teeth SSM of a target that is corrupted with missing teeth and artifacts, and the segmentation problem, which aims to indicate on the reference SSM which teeth are missing. Unlike previous methods, we address the two problems at the same time by alternating between a reconstruction step and a segmentation step. We also avoided intensity-based descriptors of individual teeth and separate tooth reconstructions by performing shape reconstruction of a full mandible SSM, removing dependence on imaging modality. Finally, the mandible SSM with teeth is built from less than 10 examples and augmented for flexibility with Gaussian kernels based on Gaussian Process Morphable Models, which avoids relying on a large annotated dataset for initial training.

## Materials and methods

### Study population and scanning protocols

For this study, we acquired a total of 76 skulls from PMCT scans (40 male, 36 female, age range: 1 to 84 years with an average age of 48.7 and a standard deviation of 24.3) of a European population. PMCT data were obtained based on the standard protocol for postmortem imaging [16] using a Siemens Somatom Definition Flash (Siemens Healthineers, Forchheim, Germany) Dual Source CT and the following parameters: slice thickness, 0.6 mm and increment 0.4 mm at 120 kV(p) with Care Dose 4D (Siemens Healthineers). The scans were all treated with the same image processing steps using the open-source software Scalismo with vtk-based thresholding, mesh extraction and smoothing, and decimation. These skulls were randomly and retrospectively selected from full-body PMCT datasets from routine cases of the Zurich Institute of Forensic Medicine. Sex was obtained from the autopsy reports. Cases of all ages were included as well as dentition with dental fillings; inlays; crowns, bridges, and toothless jaws; and implants. Individuals with fragmented jaws were excluded. To train the model, only mandibles with complete dentition and without any dental fillings, endodontic treatments, crowns, or bridges were used.

**Fig. 1:**
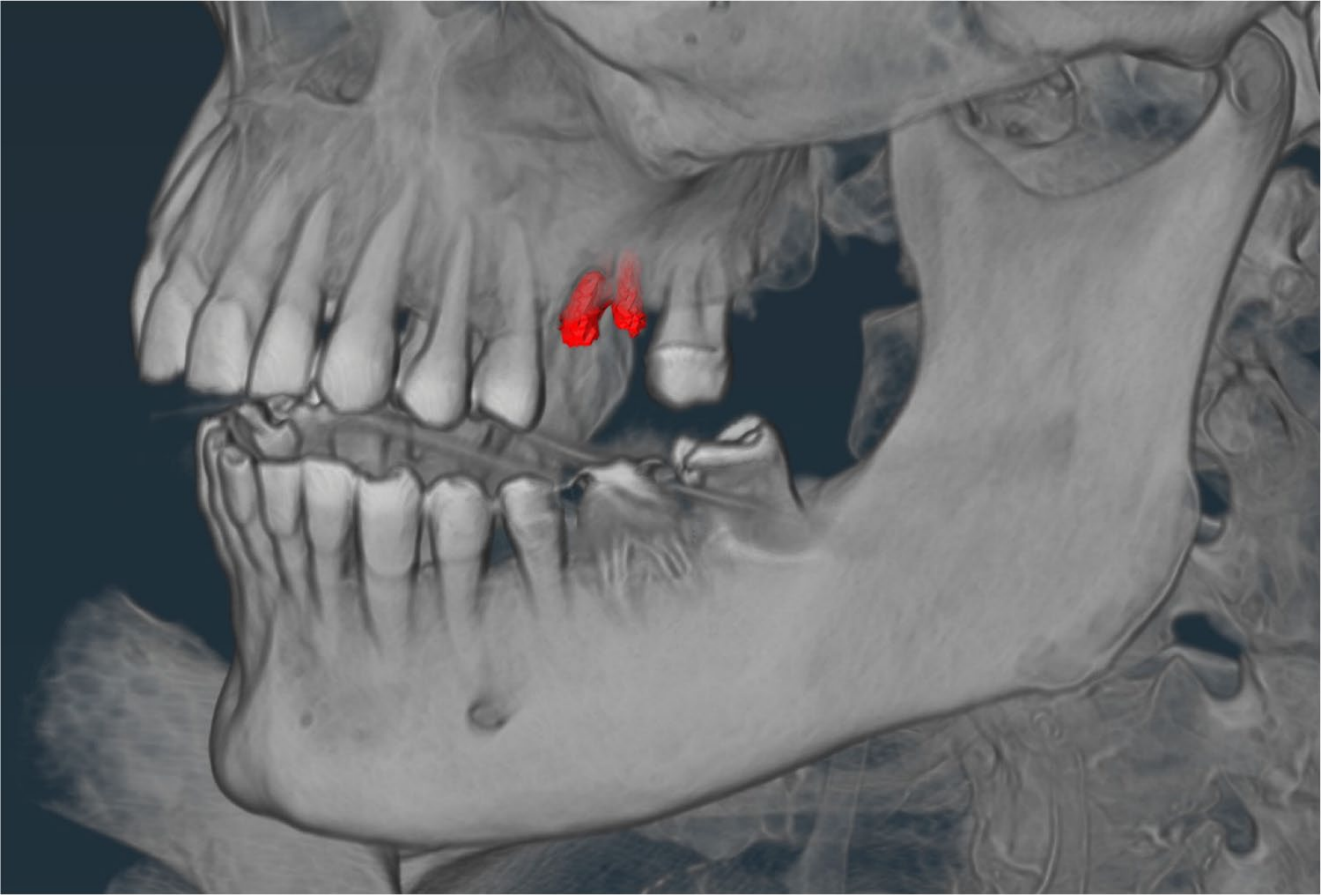
Example of root remnant (red mark)

**Fig. 2:**
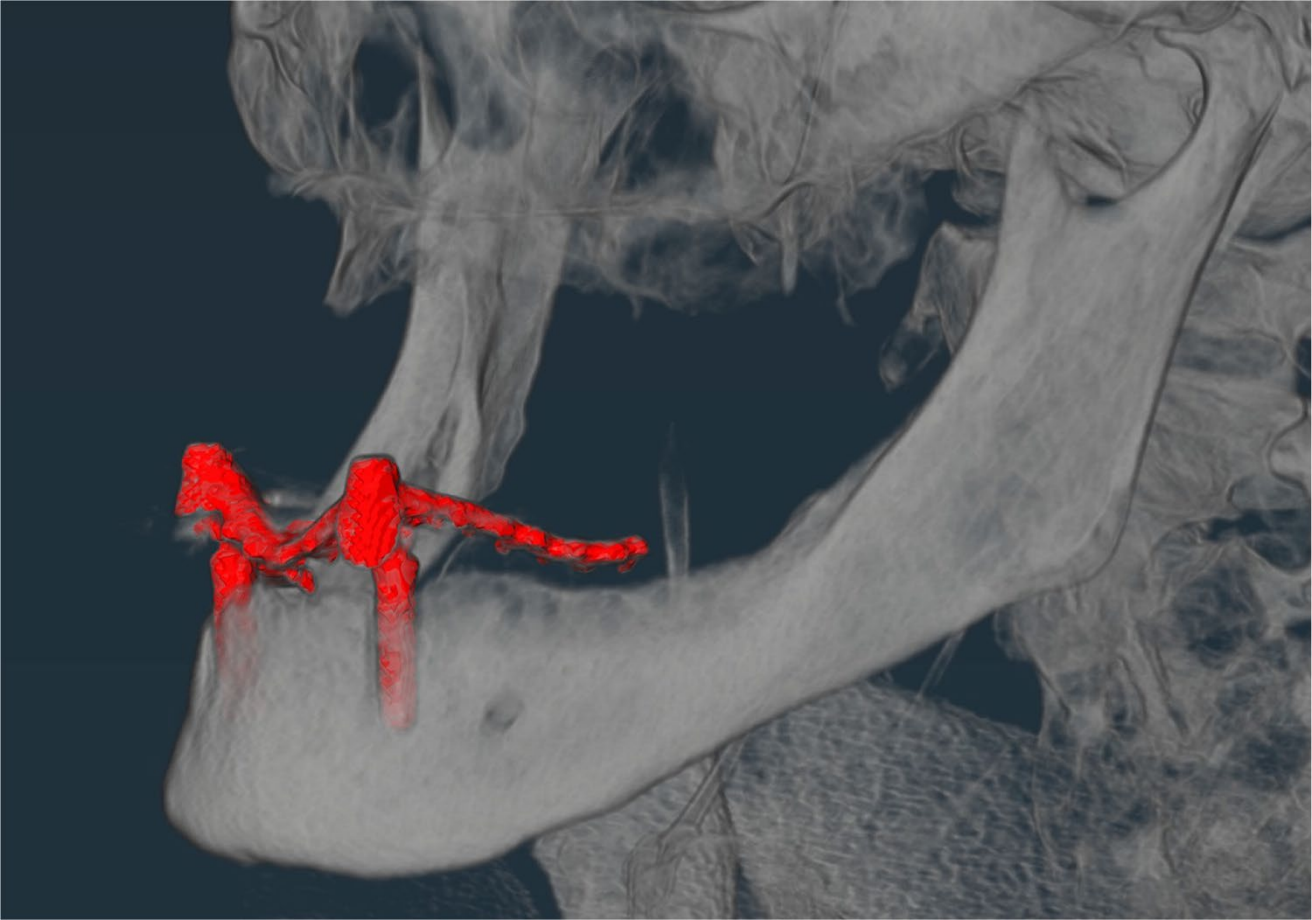
Example of an implant (two implants in the lower jaw)

**Fig. 3:**
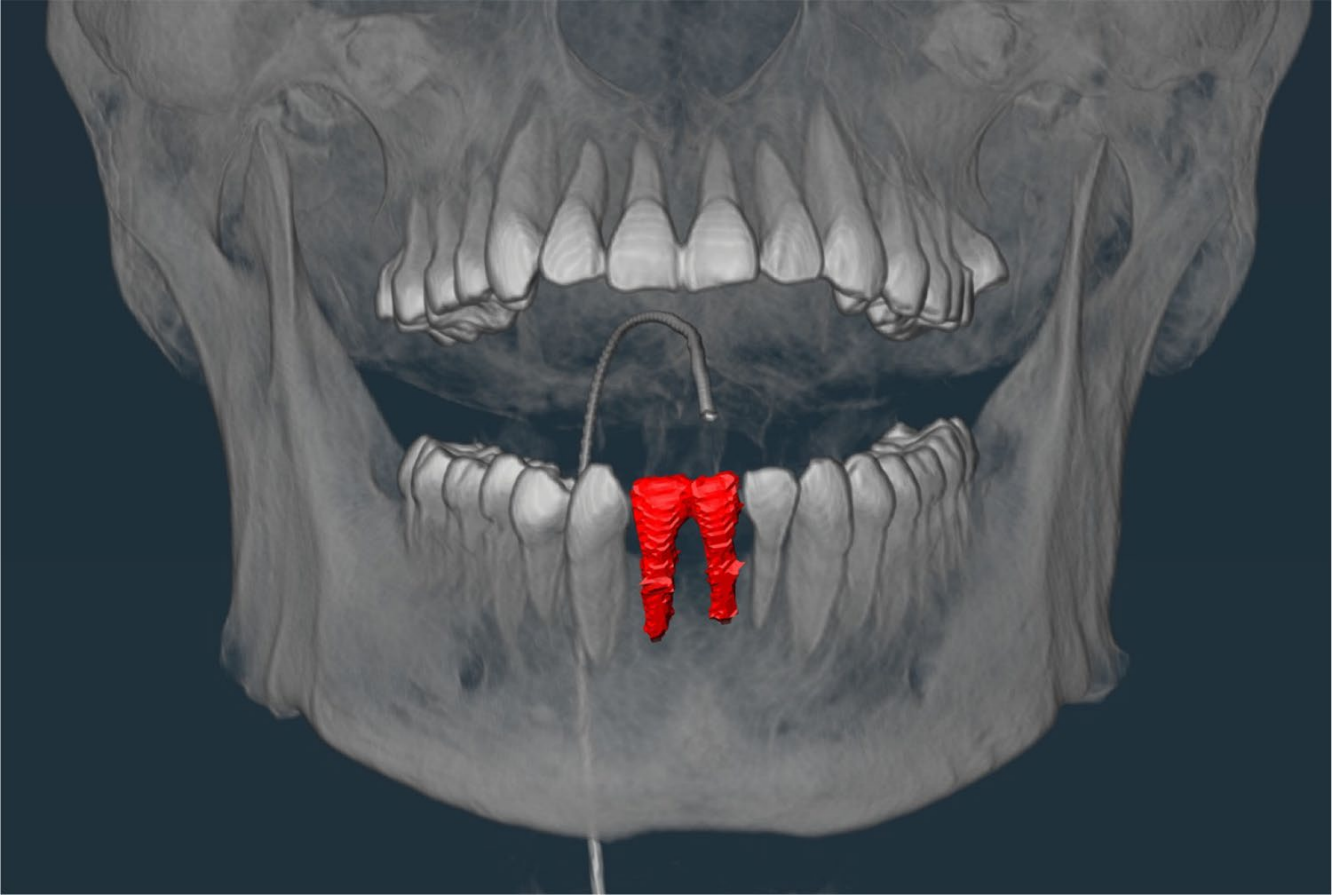
Example of gap closure (red mark at tooth 41)

**Fig. 4:**
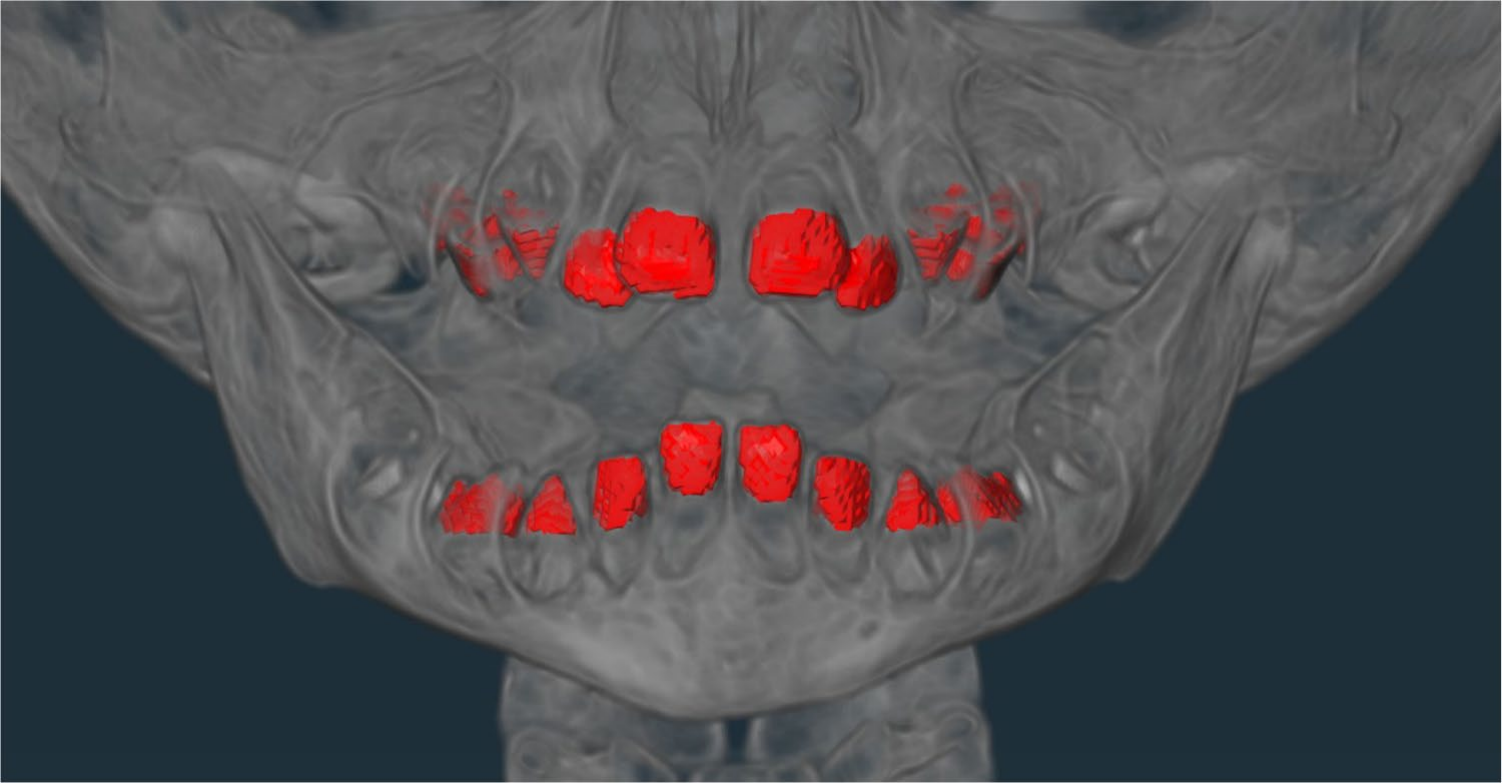
Example of first dentition

The manifestation of the teeth was made by a trained forensic dentist and divided into the following categories: 0 = tooth absent and 1 = tooth present. Other categories were added to account for exceptional findings that could not be primarily assigned to either of the other two categories, including the following dental findings: root remnant = lower part of the tooth remaining in the bone, implant = artificial tooth root, gap closure = closure of the adjacent teeth due to tooth loss in the created gap, and first dentition = deciduous teeth (children). Examples of the exceptions are shown in Figs. 1, 2, 3 and 4.

### Statistical shape models

A statistical shape model (SSM) represents the shape family in terms of the mean and the most common deformities seen in the examples. In this paper, the shape family under study is the full mandible with teeth. From this point on, we will use the mandible shape to explain SSMs and the proposed solution.

First, we will explain how SSMs are built. We refer to the standard pipeline that we used to build the mandible SSM [17]. Mandible meshes are first extracted from a set of segmented 3D CT images. The meshes are then aligned and brought into correspondence in a step called registration. Registration matches landmarks on the meshes to each other such that they are in correspondence, a necessary assumption for PCA. The correspondence assumption ensures that every landmark on the model can be matched to its landmark on a new shape instance. For example, the point located on the center of the left wisdom tooth on one mesh corresponds to the center of the left wisdom tooth on another mesh.

**Fig. 5:**
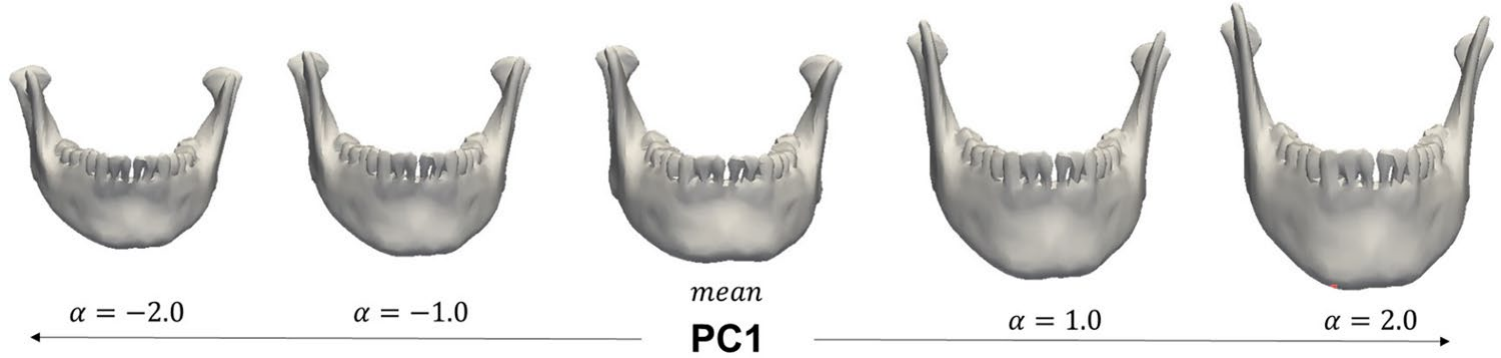
SSM mean and deformation along the first principal component deformation direction. As the value of the weight $\alpha$ moves further away from its zero-mean, the shape deforms more significantly in the same direction. Shape reconstruction is the process of finding the parameters ${\alpha }_{i},...{\alpha }_{r}$ of the principal components $\overrightarrow{P{C}_{1}},..., \overrightarrow{P{C}_{r}}$ such that the deformed shape best matches a new target

The registered meshes can now be used to build the shape model using principal component analysis (PCA). PCA learns the most common deformation directions available in the dataset from corresponding points. Deformations along the first principal component for the mandible dataset are shown in Fig. 5. This is formally written as$\overrightarrow{u }= \overrightarrow{\mu }+{\sum }_{i=1}^{r}{\alpha }_{i}\sqrt{{\lambda }_{i}}\overrightarrow{P{C}_{i }}$, where the deformation vectors obtained from PCA are$\overrightarrow{P{C}_{i }}$, their importance is indicated by eigenvalues${\lambda }_{i}$, and the first $r$ directions are used. The weights${\alpha }_{i}$,…,${\alpha }_{r}$ control the magnitude of each deformation direction. Once the SSM is built, a new instance from the same shape family can be generated by changing the weights${\alpha }_{i}$,…,${\alpha }_{r}$. Formally, it is the result of the linear combination of the mean $\overrightarrow{\mu }$ and the weighted deformations${\alpha }_{i}\sqrt{{\lambda }_{i}}\overrightarrow{P{C}_{i}}$. When the weights are zero, the mean of the model is obtained. Larger weights result in samples that are further away from the mean, as seen in the edge cases in Fig. 5. To ensure that the samples remain representative of the shape family and avoid large deformations not seen in the population, each weight is modeled as a sample from a standard normal distribution. Relying on a distribution also makes it possible to calculate the likelihood of a sample using its specific combination of weights.

To perform tooth detection, we rely on a mandible SSM. The mandible SSM is built from 9 full mandible meshes that are different from the test set. The mandibles used for model building are not missing any teeth and include the wisdom teeth and condyles.

### Simultaneous shape reconstruction and label map prediction

Given a new mandible shape, the model is fitted to the target in a process called shape reconstruction. During shape reconstruction, the weights ${\alpha }_{i}$…,${\alpha }_{r}$ are modified until the deformed shape from the SSM matches the target shape. The quality of the match is determined using the reconstruction loss, which is the average Euclidean distance between the model vertices and their corresponding target vertices. In this paper, we rely on a publicly available iterative reconstruction algorithm based on Gaussian process regression to obtain the weights ${\alpha }_{i}$…,${\alpha }_{r}$ [17].

When presented with target mandibles that have missing teeth, the correspondence assumption is not satisfied. This is because some teeth present on the model are not present on the target. Furthermore, the missing teeth are not labeled in advance. Therefore, in addition to the reconstruction problem, there is the problem of detecting missing teeth. Two problems must therefore be solved simultaneously because their results influence each other: obtaining the missing teeth labels and obtaining the shape reconstruction.

To address this problem, we opt for a region-growing reconstruction strategy with an open-source implementation [18]. The strategy takes as input some landmarks on the target that indicate healthy regions. We chose 10 landmarks distributed on the mandible as follows: two on each condyle (4 landmarks in total), one on each coronoid (2 landmarks in total), one on each wisdom tooth (2 landmarks in total), one on the interdental papilla between tooth number 31 and tooth number 41 as numbered by the FDI notation, and one on the chin. The model is updated such that the reconstruction loss at those landmarks is minimized. Given the updated shape, new healthy regions are predicted based on their Mahalanobis distance to the model under the SSM likelihood. The new healthy regions are then used to update the model shape again. The process continues until no new healthy regions are detected. At this point, all the regions on the shape that were not labeled healthy are marked as pathological. This results in a binary label map: vertices with label 1 indicating healthy and vertices with label 0 indicating unhealthy. Given this label map, we propose an additional step to predict the missing teeth.

### Proposed solution for tooth detection from label map

The binary label map obtained is used in this proposed teeth detection step. In our case, the mandibles are expected to have missing teeth compared to the healthy reference shape. All the obtained pathological regions were used to predict which teeth were missing.

First, we label each tooth region on the mean shape of the SSM. This labeling only needs to be performed once on the mean shape and then can be detected automatically on the reconstructed shape because of the correspondence assumption.

**Fig. 6:**
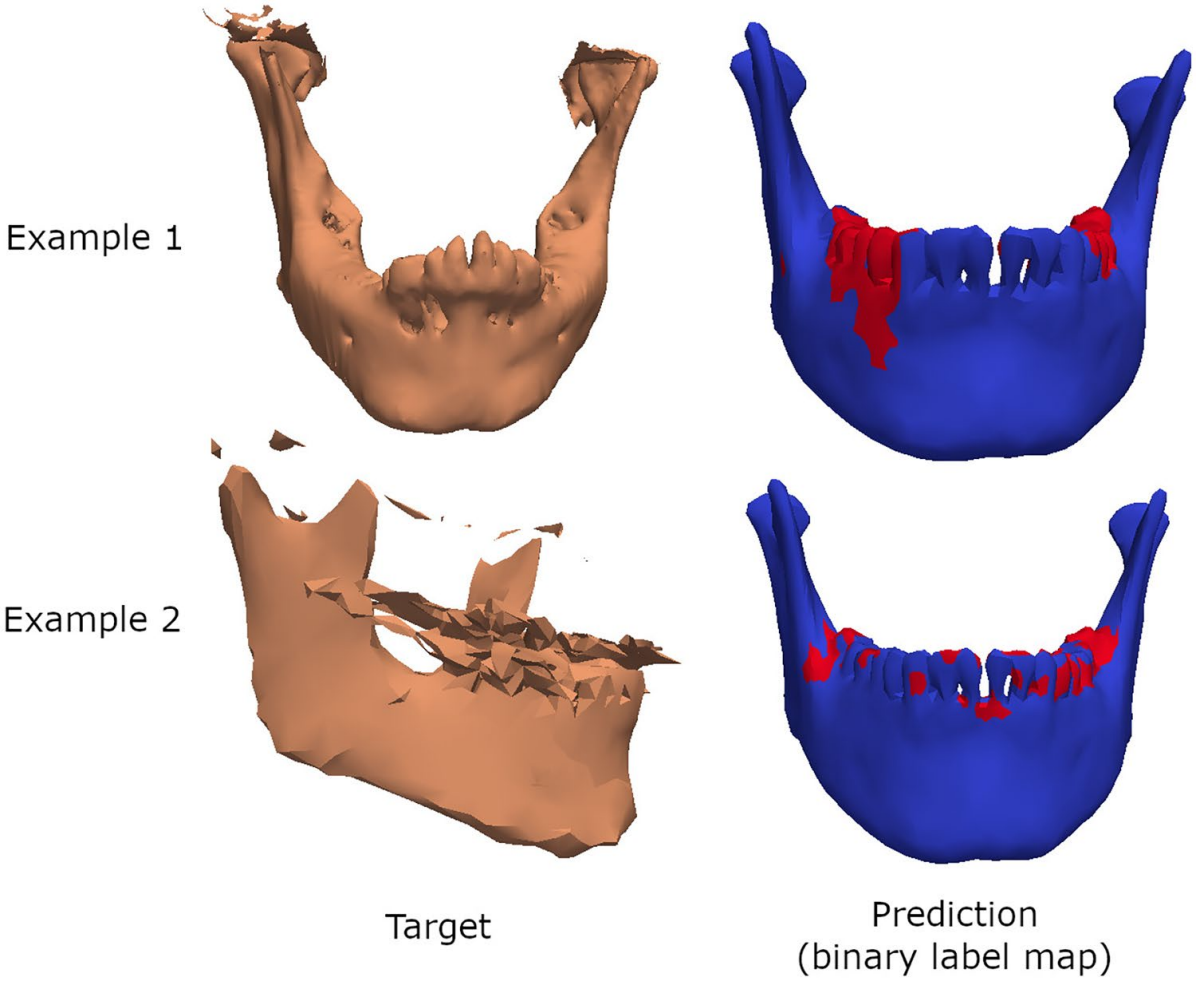
Two example target mandibles alongside their binary label map predictions. The label map indicates in red the regions that have unlikely deformations and in blue the healthy regions. If more than 70% of a tooth has a red label in the binary label map, then that tooth is classified as missing

Using the predicted label map from step 2 and the labeled tooth regions on the mean of the SSM explained above, the missing tooth prediction step can take place. In Fig. 6, the label maps of two example target mandibles are visualized for reference. We evaluate the binary label map at each tooth region. If 70% of the vertices associated with that tooth have an unhealthy label in the predicted binary label map, then that tooth is labeled missing; otherwise, it is labeled present. We repeat this process for every tooth in the mandible: teeth 31 to 38 and then again 41 to 48, as numbered by the FDI notation. The predictions are compared to the ground truth annotations.

## Results

**Fig. 7:**
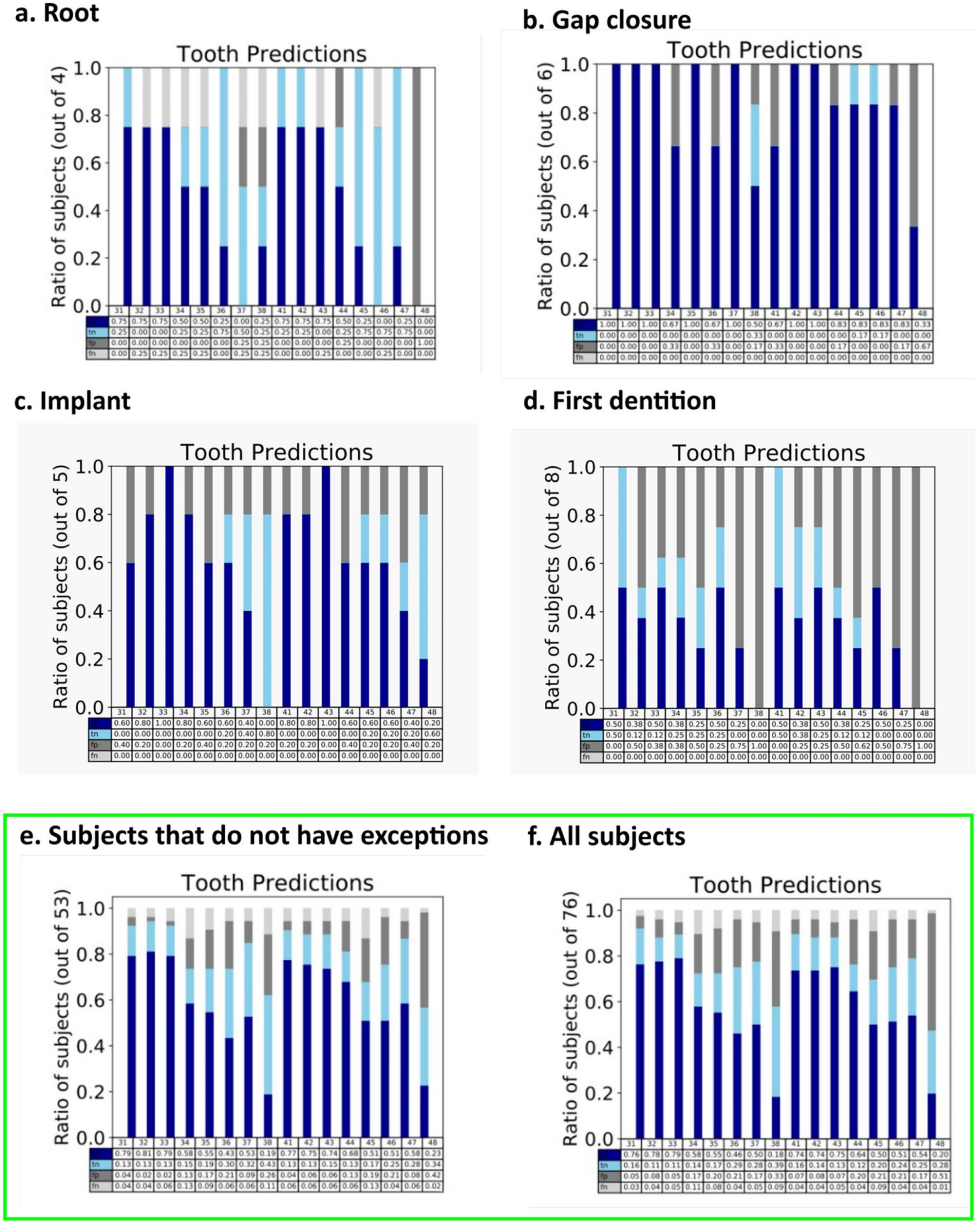
Method performance for different exceptions for different teeth. Dark blue, true positives (a present tooth is detected); light blue, true negatives (tooth marked as missing when actually missing); dark gray, false positives (tooth marked as present when actually missing); and light gray, false negatives (tooth marked as missing when actually present). Exceptions are as follows: **a** root (*n* = 4), **b** gap closure (*n* = 6), **c** implant (*n* = 5), and **d** first dentition (*n* = 8). In addition, all cases without exceptions (**e** *n* = 53), as well as a combination of all cases (**f** *n* = 76), are visualized

The average accuracy of tooth detection over all teeth using statistical shape modeling was 0.77 for the case group with exceptional findings (Group 1) and 0.78 for the case group excluding exceptional findings (Group 2). The accuracy, however, differs from tooth to tooth, with incisor detection having the greatest accuracy with 0.92 (Group 1) and 0.92 (Group 2) and decreasing accuracy the further back the teeth lie, dropping to 0.47 (Group 1) and 0.56 (Group 2) for the wisdom teeth. The presence of scans from the exception group had almost no influence on the accuracy, with the mean accuracy error when comparing the outcome between both groups being 0.03 (Table 1). In addition, both groups were strongly correlated (Pearson’s correlation coefficient *r* = 0.95). A summary of the findings, including the performance for different exceptions, can be seen in Fig. 7.

**Table 1:**
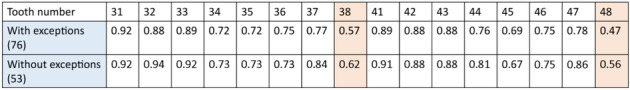
Accuracy of dental detection per tooth including exceptions (root, gap closure, implant, first dentition (top row, 76 cases)) and excluding exceptions (bottom row, 53 cases)

## Discussion

In this study, we proposed using statistical shape modeling for identifying the dentition of the mandible and detecting missing teeth automatically.

The results show that the method is more accurate in the frontal part of the mandible, where accuracy rates were approximately 90%, than in the posterior part of the mandible. As the statistical shape modeling is based on the shape of the tooth and not the intensity, this result can be explained by the smooth transition of the wisdom teeth into the jaw without significant shape differences. This explains the false-positive seen in Fig. 7e for teeth numbers 38 and 48, where the presence of a tooth was falsely detected. In practice, the shape model exhibits shrinking, whereby teeth 38 and 48 in the SSM are incorrectly fitted to the jaw region. This can be resolved by including partial shape models for the molars, more landmarks that mark the transition from tooth to jaw regions, or other descriptors that can be used to indicate the transition.

Comparing our results to previous studies for labeling teeth in CT scans, it is obvious that the wavelet-Fourier method used by Miki et al. achieved a higher accuracy than the proposed solution [19]. Furthermore, the high false-positive rates indicate the difficulty of recognizing tooth gaps with this approach. This can be explained by the fact that tooth extraction causes a shift in dentition over time, resulting in gap closure. This seems to be a problem, especially for molars, as they have quite similar shapes and are the most commonly extracted teeth [20, 21], which becomes evident with the low accuracy of our approach at the molars.

However, other approaches require specific imaging conditions. For example, Miki et al. used scans performed in a clinical setting in an open bite condition, which is a scenario rarely found in postmortem imaging [11, 19]. Due to rigor mortis or other taphonomic conditions, neither an open bite condition nor an alignment of the head in an anatomically straight position can be achieved. Furthermore, the authors stated that metallic artifacts due to implants or fillings were a major obstacle in teeth labeling. These requirements highlight the flexibility of our proposed solution, which can be applied in practical circumstances and does not require specific imaging environments or target conditions. For example, comparing the cases without any dental work and with fillings, implants, or crowns in our study shows no significant differences between the groups. In contrast to the other studies, we also included extreme cases, such as children with deciduous teeth. In addition, the presence of ventilation tubes does not impact our algorithm because of the specificity of the SSM, which generates new samples of likely mandibles. Statistical shape modeling was previously used by Duy et al. to differentiate between existing or missing teeth of the maxilla [14]. Similar to our results, they found no difficulties with teeth with metallic implants. This is because SSM only uses the shape difference of the teeth for detection and not the density. For example, cases with decomposition can still be addressed with the proposed solution. However, the datasets only contained two cases with ventilation tubes, which limited drawing any further conclusions about the significance of the results.

Focusing on the shape rather than the intensity of the tooth, statistical shape modeling could also be applied to other 3D modalities (surface scan or magnetic resonance imaging (MRI)). This allows a wider range of possible antemortem data usage in identification processes. Even though fractured mandibles were excluded from this study, the method can work with bone fragments as well, allowing for the reconstruction of the mandible with complete dentures before performing tooth detection. Such an approach has already been presented for the reconstruction of other target organs, such as the femur [22], where the reconstruction was used as a proxy of the full shape for measurements that should be taken on the missing parts. Future research should test this model on fragmented jaws to test the applicability of the model while dealing with these circumstances in mass disaster scenarios.

Overall, this method seems to be applicable in DVI scenarios when dealing with a high number of decomposed and mutilated bodies.

## Conclusion

The application of SSM for automatically detecting missing teeth showed a lower accuracy for the teeth in the back of the jaw, which could be a disadvantage for use in forensic cases.

On the other hand, the method we used is not impaired by dental work or medical installations such as ventilation tubes. We also propose that it is applicable to fractured mandibles as well as to other imaging modalities, which makes positive identification more possible when implemented in mass fatality scenarios.

## Key points

1. The study results propose the use of statistical shape modeling for identifying the dentition of the mandible and detecting missing teeth automatically.
2. The results show that the method is more accurate in the frontal part of the mandible, where accuracy rates were approximately 90%, than in the posterior part of the mandible.
3. The proposed solution is flexible, can be applied in practical circumstances, and does not require specific imaging environments or target conditions, such as an open bite condition or alignment of the head in an anatomically straight position.

## Data Availability

The data from this study were part of the doctoral thesis paper from D.R. Data can be seen in Table 1 and Fig. 7.
